# Development and characterization of genomic resources for a non-model marine teleost, the red snapper (*Lutjanus campechanus*, Lutjanidae): Construction of a high-density linkage map, anchoring of genome contigs and comparative genomic analysis

**DOI:** 10.1371/journal.pone.0232402

**Published:** 2020-04-29

**Authors:** Adrienne E. Norrell, Kenneth L. Jones, Eric A. Saillant

**Affiliations:** 1 School of Ocean Science and Engineering, Gulf Coast Research Laboratory, University of Southern Mississippi, Ocean Springs, MS, United States of America; 2 Department of Biochemistry and Molecular Genetics, University of Colorado School of Medicine, Aurora, CO, United States of America; Universite de Lausanne Faculte de biologie et medecine, SWITZERLAND

## Abstract

The red snapper *Lutjanus campechanus* is an exploited reef fish of major economic importance in the Gulf of Mexico region. Studies of genome wide genetic variation are needed to understand the structure of wild populations and develop breeding programs for aquaculture but interpretation of these genome scans is limited by the absence of reference genome. In this work, the first draft of a reference genome was developed and characterized for the red snapper. P-454 and Illumina sequencing were conducted to produce paired-end reads that were assembled into reference contigs and scaffolds. The current assembly spans over 770 Mb, representing an estimated 69% of the red snapper genome in 67,254 scaffolds (N50 = 16,803 bp). The genome contigs were applied to map double digest Restriction-Site Associated DNA Tags and characterize Single Nucleotide Polymorphisms (SNPs) in five outbred full-sib families. The identified SNPs and 97 microsatellite loci were used to generate a high-density linkage map that includes 7,420 markers distributed across 24 linkage groups and spans 1,346.64 cM with an average inter–marker distance of 0.18 cM. Sex-specific maps revealed a 1.10:1 female to male map length ratio. A total of 4,422 genome contigs (10.5% of the assembly) were anchored to the map and used in a comparative genomic analysis of the red snapper and two model teleosts. Red snapper showed a high degree of chromosome level syntenic conservation with both medaka and spotted green puffer and a near one to one correspondence between the 24 red snapper linkage groups and corresponding medaka chromosomes was observed. This work established the first draft of a reference genome for a lutjanid fish. The obtained genomic resources will serve as a framework for the interpretation of genome scans during studies of wild populations and captive breeding programs.

## Introduction

Studies of the genetic architecture of population adaptations or of the genetic basis of complex characters in non-model species have been limited by the high costs and efforts involved in generating reliable reference genomes [1]. The production of a fully assembled reference genome still requires a considerable investment of resources in species with large genomes, but partial genome assemblies with relatively high coverage can be attained at a reasonable cost using high-throughput sequencing technologies. These partial sequences are often fragmented and can contain misassembled regions [2], making their direct application to applied genomic studies challenging. However, the utility of these assemblies is greatly enhanced if they are anchored to a high-density linkage map [2].

This anchoring is achieved by mapping contigs and scaffolds onto linkage groups to generate pseudo-chromosomes [3]. The mapping and ordering of contigs and scaffolds contributes to the genome sequence assembly process by allowing putative assembly errors to be identified and corrected [2,4]. Mapped contigs can also be applied in comparisons of genome organization among species [5] [6] [7]. Another potential application of the integrated map and genome sequence is in the interpretation of high density genomic scans during population genomic surveys. Polymorphisms identified and surveyed during these studies can be mapped on genome contigs which allows positioning them on linkage groups and inferring their degree of linkage. The information gathered is critical when inferring genomic regions involved in divergence and the genomic architecture of local adaptations [8] [9] or when estimating effective population size and recent demographic growth trajectories of populations [10]. Finally, mapped genome contigs can also be used in Quantitative Trait Loci (QTL) mapping studies aiming to locate loci impacting phenotypic characters affecting fitness or traits of importance to commercial production in aquaculture species, further contributing to the comprehensive characterization of the genetic basis of these traits [11].

The objective of this work was to initiate the development of genomic resources for the red snapper (*Lutjanus campechanus*), a marine reef-associated fish belonging to the Lutjanidae family. The species is exploited by major fisheries throughout its range and is currently being developed for aquaculture in the United States. Several studies investigating red snapper population structure in recent years [12] [13] [14] have aimed to define management units to better conserve fisheries resources. These early studies were based on small numbers of genetic markers and failed to assess the role of local adaptation in structuring red snapper populations or to document the genetic basis of significant regional differences in several life history traits [15,16]. More recently, genome scans generated by double-digest RAD sequencing were deployed to study fine scale genetic structure among juveniles and adults [17] in the northcentral Gulf of Mexico. Expanding the use of genome scans for populations genetic studies towards analysis of adaptive variation, and their application to domestication programs in red snapper is warranted but, as discussed above, their interpretation is currently limited by the lack of reference genome for red snapper or any other lutjanid. This work aimed to anchor the first draft of a reference genome sequence with a high-density linkage map for red snapper and provide a first comparison of the organization of the red snapper genome with that of other teleosts.

## Materials and methods

### Permits and ethic statement

Wild red snapper used in the study were collected under the letter of acknowledgment S-12-USM/GCRL-GOMESA of the U.S. National Marine Fisheries Service. Protocols used in this study were developed with the guidance of the Institutional Animal Use and Care Committee of the University of Southern Mississippi and approved by the committee (protocol # 10100108). In compliance with this protocol, specimens that were sacrificed for sequencing were euthanized by immersion in a lethal dose (400ppm) of MS-222 anesthetic.

### Genome sequencing and assembly

Genomic DNA was extracted with the Qiagen DNeasy Blood & Tissue Kit from the spleen of one wild red snapper individual collected from Alabama (U.S.) coastal waters. One microgram of the obtained DNA was used to prepare an Illumina V3 whole-genome shotgun Paired-End library according to manufacturer protocol. The library was size selected to retain 300 bp fragments and sequenced on one flow-cell lane of the Illumina HiSeq2000 platform at the University of Colorado Denver School of Medicine (Aurora, CO, USA). Another 35 μg of the same DNA was used to generate 3 kb and 20 kb Paired-End libraries for sequencing on the Roche P-454 GS-FLX titanium platform at the IGSP Genome Sequencing & Analysis Core Facility at Duke University (Durham, NC, USA). The libraries were loaded on one PicoTiterPlate for sequencing, half of which was allocated to the 3 kb library and the other half to the 20 kb library. Illumina sequencing reads were trimmed and filtered to retain reads with at least 75 bp and Phred scores greater than 20.

The size of the red snapper genome was estimated using the Jellyfish k-mer counting program [18]. The frequency distribution of k-mer depth obtained from Jellyfish was used to determine the mean coverage estimated as the mode of the Poisson distribution after trimming to remove reads with random sequencing errors. Genome size was estimated as the total number of k-mers divided by the mean coverage.

The sffToCA module of the Celera Assembler version 8.0 [19] [20] was used to prepare P-454 reads for assembly under the parameters ‘clear n’ and ‘trim hard’. Filtered sequencing reads were assembled with the Celera Assembler. All reads were used in the assembly of contigs and mate pair information was used to split contigs when read coverage pattern indicated a unitig had formed with a chimeric read. Mate pair information from P-454 and illumina paired reads and unitigs obtained from the contig assembly process were used in scaffolding. Contigs consisting of fewer than 200 bp were discarded from the final assembly. A search of the NCBI NT database was performed using BLASTn (e-value 1e-06) to identify bacterial sequences erroneously included in the assembly; if one or more of the top 3 BLASTn hits had a bacterial origin, the contig was removed [21].

### Mapping population

Wild adult red snapper (5 males and 5 females) collected from Alabama coastal waters in 2012 and 2013 were used to generate 5 full-sib families at the University of Southern Mississippi’s Thad Cochran Marine Aquaculture Center. Each family was produced by manually stripping the ova from one female and fertilizing them *in vitro* with the sperm of one male as described in Minton et al. [22]; ovulation of females and spermiation of males had been induced by a single injection of human chorionic gonadotropin (1,100 IU.kg^-1^ for females, 550 IU.kg^-1^ for males). Larvae were reared for 60–90 days before tissue collection to ensure a sufficient amount of DNA could be obtained using non-lethal sampling techniques. Tissue clips were taken from the dorsal fin of each fish and immediately immersed in a 20% DMSO salt-saturated fixative (0.5 M EDTA, 20% Dimethyl sulfoxide, NaCl, ddH_2_O) for preservation. Whole genomic DNA was extracted from each parent and 60 randomly selected offspring per family using the phenol-chloroform protocol [23]. In Family 2, only 51 full-sib samples could be obtained. DNA quality was evaluated on a 1% agarose gel; samples displaying minimal apparent DNA degradation were assessed via spectrophotometry on a Nanodrop 2000 (Thermo Fisher Scientific, Waltham, MA, USA) to determine DNA concentration and purity. High quality DNA samples were adjusted to 50–70 ng.μl^-1^ with Tris-HCl (10 mM, pH 8.0) and stored at -20°C until use.

### Microsatellite assays

Each individual was genotyped at 97 microsatellite markers. Eighty-one of the microsatellites were developed by Norrell et al. [24] and optimized into multiplexes consisting of 2 to 4 markers. The remaining 16 microsatellites were characterized by Gold et al. [25] and combined into multiplex assays by Renshaw et al. [26]. Polymerase Chain Reactions (PCR) were completed in a total volume of 5 μl, consisting of 4.8–7.2 ng of genomic DNA, 2.5 pmol of forward and reverse primers, 10 nmol of MgCl_2_ (Promega, Madison, WI, USA), 1 nmol of dNTPs (Promega), 0.5 units of GoTaq Flexi DNA Polymerase (Promega) and 1x of buffer (Promega). The thermal cycling protocol used for PCR began with a denaturation step at 95ºC for 3 min, followed by 35 cycles at 95ºC for 30 s, annealing temperature for 30 s and 72ºC for 45 s. Amplification finished with an elongation step at 72ºC for 10 min. The annealing temperature (AT) used for amplification was optimized based on the specific AT of the microsatellites markers incorporated in multiplex panels. When the specific AT differed among markers included in the same multiplex panel, a touchdown thermal cycling protocol was used where the AT was gradually reduced during consecutive annealing cycles as described in Renshaw et al. [26]. The composition of multiplex panels and ATs used for multiplex amplifications are available in the supplementary materials (S1 Table). PCR products were loaded on an ABI-377XXL sequencer (Applied Biosystems, Carlsbad, CA, USA) and the resulting electropherograms were analyzed with GeneScan (Applied Biosystems) software. Allele calling was performed using Genotyper (Applied Biosystems) and genotypes were entered into an excel spreadsheet. Parents and progeny of the same full-sib family were run on the same gel and analyzed together. The program LINKMFEX v. 3.1 [27] (available at http://www.uoguelph.ca/~rdanzman/software.htm) was applied to test for segregation distortion; loci deviating significantly from the expected Mendelian segregation ratio (*P* < 0.05) were removed from the dataset on a per family basis.

### Double digest RAD-Tag sequencing

Double digest RAD-Tag sequencing libraries were prepared using protocols modified from Baird et al. [28] and Peterson et al. [29]. Each library was made from 0.5 to 0.9 μg of whole genomic DNA, simultaneously digested by Sau3AI (7.5 units, New England Biolabs Inc., Ipswitch, MA, USA; NEB) and SPEI (3.75 units, NEB) at 37°C for 1 h. After restriction enzymes were heat-inactivated at 65°C for 15 min, sample-specific Illumina adapters were ligated to the digested fragments using 400 units of T4 Ligase (NEB) at 16°C for 30 min. Each adapter pair included a unique 6 bp barcode, used to retrieve reads from individual samples following multiplex sequencing, and a degenerate 8 bp unique molecular identifier (UMI) that enabled the downstream identification and removal of PCR duplicates [30]. The ligated samples were purified and size-selected to remove fragments < 250 bp by applying Agencourt AMPure XP beads (Beckman Coulter Inc., Brea, CA, USA) at a 0.65x ratio. The library fragments were then amplified in 25 μl reactions consisting of 1X of *Taq* 2X Master Mix (NEB) and 5 pmol of forward and reverse primers. The thermal cycling program began with a denaturation step of 3 min at 95°C, 30 cycles of 95°C for 30 s, 55°C for 30 s and 72°C for 30 s, and ended with a final extension step at 72°C for 7 min. PCR products were pooled according to lane (31 individuals sequenced per Illumina flow-cell lane), purified with Agencourt AMPure XP beads (0.65x), and size-selected on a PippinPrep (2% agarose gel cartridge, Sage Science, Beverly, MA, USA) to include fragments between 300 and 500 bp in the final library. The eluate was purified using a 1.8x ratio of Agencourt AMPure XP beads before being submitted to the University of Colorado Denver School of Medicine for sequencing. Four families were sequenced on the Illumina HiSeq2000 platform and sequencing of the remaining family was conducted using the Illumina HiSeq2500 platform. Individuals from the same family (parents and offspring) were multiplexed in two pools that were sequenced during the same run on two sequencing lanes.

### SNP discovery, genotyping and filtering

Low confidence base calls (Phred score < 30) were iteratively removed from paired-end reads, beginning with the last base in each sequence (the script used in filtering was uploaded in the USM Aquila repository doi: 10.18785/lutc.ds.01). Both the forward and reverse reads in a pair were discarded if either fell below 75 bp in length during the trimming procedure. Identical sequences sharing the same UMI were marked as PCR duplicates; only one read from each set of duplicates was retained. Filtered reads were fed into the program dDocent [31], where remaining adapter dimers were culled from the dataset and reads were mapped against the red snapper draft genome using BWA-MEM [32]. The Bayesian variant caller FreeBayes [33] was then applied to the mapped reads to discover and genotype Single Nucleotide Polymorphisms (SNPs) and insertions/deletions (INDELs).

SNPs were selected from the obtained database by applying the following filtering procedure. First, multiallelic SNPs, INDELs, SNPs covered by fewer than 10 reads or more than 1.5X the average depth across the dataset (32.44) and SNPs having a site quality score below 30 as determined by FreeBayes were removed from the dataset. SNPs supported by both forward and reverse reads were then excluded as they potentially arose from mapping errors that occurred during SNP discovery. Individual genotypes with a likelihood lower than 0.99, as estimated in FreeBayes, were recoded as missing data (0/0). Individuals missing genotypes at more than 80% of the filtered loci were excluded from further analysis. The final dataset consisted of SNPs for which genotypes were available in at least 75% of the remaining individuals.

### Linkage map construction

Linkage analyses were conducted in the software Lep-MAP2 [34,35]. Prior to mapping, missing and incorrect parental information was imputed from progeny genotypes using the ParentCall module of Lep-MAP2 and the final dataset was filtered in ParentCall to retain SNPs showing no evidence of segregation distortion (*P* < 0.01) and that were informative in at least two families. Filtering strategies resulted in a minimum of 84 informative meioses available for linkage analysis. Increasing values of the lodLimit threshold LOD score were tested in Lep-MAP2. A LOD score of 9 recovered 24 major Linkage Groups (LGs) and was applied to assign microsatellites and SNPs to LGs. Small LGs containing fewer than 10 loci were rejected from the map. Marker ordering was then completed using the OrderMarkers module following methods described by Rastas et al. [34]. Each LG was ordered 10 times, simultaneously estimating marker-specific error rates and map distances based on the Kosambi function [36] in males and females. Error prone loci (error rate > 0.1) and markers inflating the ends of linkage groups by more than 10 cM were removed and the order was re-evaluated an additional 10 times [34]. The order with the highest likelihood was selected from this set and final sex-averaged map lengths were determined. The lengths of individual LGs were summed to calculate the total length of the map. MapChart [37] was used to visualize the linkage map.

The program ALLMAPS [3] was then used to order and orient the corrected contigs against the sex-averaged linkage map. Mapped SNPs anchored the contigs, forming putative red snapper pseudo-chromosomes. When contigs contained multiple markers, their mapping positions were used to orient the segment. The orientation of contigs anchored by only one SNP could not be determined.

### Genome comparisons

The genome assemblies of medaka (*Oryzias latipes*, v. ASM31367v1) and spotted green puffer (*Tetraodon nigroviridis*, version 8.0) available in the Ensembl genome database were used for comparison to the red snapper. Repeat-masked assemblies were obtained as individual chromosome files which were concatenated into one multi-entry FASTA file per species. Red snapper contigs and mini-scaffolds anchored to the linkage map were screened for repetitive elements in RepeatMasker v. 4.0.7 [38] and detected repeats were hard-masked. Masking employed the interspersed repeat masking based on protein similarity option of repeatmasker. Pairwise comparisons were then performed between the red snapper assembly and the genomes of the two reference species. Syntenic regions were detected using BLASTn (v.2.10.0+) using default parameters (e-value 1e-10). If a segment aligned to multiple areas within a model organism, only the best hit was retained. The distributions of aligned red snapper sequences among the chromosomes of each of the target species were plotted as Oxford grids in excel software (v. 2016).

## Results

### Draft reference genome assembly

Assembly of P-454 and Illumina sequencing reads yielded 76,345 contigs larger than 200 bp in length. All contigs were screened for bacterial contamination in the NCBI NT database; 124 contigs possibly included bacterial sequences and were removed from the assembly, bringing the total number of contigs to 76,321. The assembly spanned 770,603,859 bp. Estimates of the size of the red snapper genome by the k-mer method were similar for k values ranking between 15 and 31bp and averaged 1.117 Gb (range 1.070 Gb-1.126 Gb). Based on this estimate, the assembly would span approximately 69% of the genome. Contig length ranged from 200 to 157,915 bp with a mean of 9,928 bp and the assembly had an N50 value of 14,414 bp. Paired-end information allowed assembling a total of 67,254 scaffolds (N50 = 16,803 bp; average 1.15 contigs per scaffold). The average contig coverage of the assembly was 24.37X. Raw sequencing reads and assembled contigs were uploaded to the SRA NCBI database and can be accessed under the BioProject number PRJNA325063 (P-454 reads: SRX1835988, Illumina reads: SRX1835987, contigs: https://trace.ncbi.nlm.nih.gov/Traces/sra/sra.cgi?analysis=SRZ190074, scaffolds: https://trace.ncbi.nlm.nih.gov/Traces/sra/sra.cgi?analysis=SRZ190075).

### Microsatellite genotyping

Genotypes at the 97 microsatellites were acquired for the 291 progeny and the 10 parents involved in the crossing design. Five of the loci could not be scored with confidence in one or more families due to stuttering; the corresponding data (7 family by microsatellite marker combinations) were discarded from the dataset. In 19 of the remaining 478 family by marker combinations, some progeny genotypes differed from expectations under Mendelian transmission laws. Examination of parent and offspring genotypes revealed that these disagreements corresponded to cases where one of the parents was scored homozygous but several progeny failed to display the allele that this parent was expected to transmit, leading to an apparent Mendelian incompatibility. It was concluded that the homozygous parent was the carrier of a non-amplifying null allele. When the progeny genotypes could be unambiguously determined by accounting for the null allele, their genotypes were recoded with the null allele and the marker was retained. However, in cases where the two parents shared an allele (3 family by marker combinations), differentiating between progeny carrying a null allele and true homozygous individuals was not possible and the data were discarded. Significant departures from Mendelian segregation ratios were detected in 28 family by marker combinations; the corresponding data were discarded from further analysis. The final dataset included 76 to 96 microsatellites per family and was applied to the construction of the linkage map (Table 1).

**Table 1:** Number of filtered sequencing reads, microsatellites and SNPs included in family datasets. Average read depths and mapping qualities across SNPs for each family are shown.

	Family 1	Family 2	Family 3	Family 4	Family 5
Sequencing Reads (M)	127.3	110.8	210.4	200.7	216.9
Microsatellites	90	95	96	90	76
Number SNPs	9061	8716	27118	13569	10331
Average Read Depth	20.71	21.70	25.52	24.50	22.40
Average Mapping Quality*	58.64	58.91	59.05	59.42	59.21

* Mapping quality scores generated in BWA

### RAD-sequencing, SNP discovery and genotyping

Double digest RAD-tag sequencing produced over 866 M reads across 5 families. The number of filtered reads per individual ranged from 4 k to 9.27 M, with an average of 1.73 M (family totals are reported in Table 1). After filtering raw variants identified by FreeBayes for polymorphism, genotype quality and sample size, 8,716 to 27,118 candidate biallelic SNPs remained per family (Table 1). Fifteen individuals were discarded due to poor genotyping success. On average, SNPs were supported by between 20.71 and 25.52 reads across families (Table 1). Average mapping qualities generated in BWA-MEM for reads used in SNP genotyping were consistent between families, ranging from 58.64 to 59.42 (Table 1). The final dataset consisted of 10,150 SNPs informative in 2 or more families available per SNP locus for linkage analysis. A variant call format file describing all the markers and a table reporting the genotypes and pedigrees of individuals (parents and offspring from each family) are available on the University of Southern Mississippi public repository Aquila (DOI: 10.18785/lutc.ds.01).

### Linkage map construction

**Fig 1:**
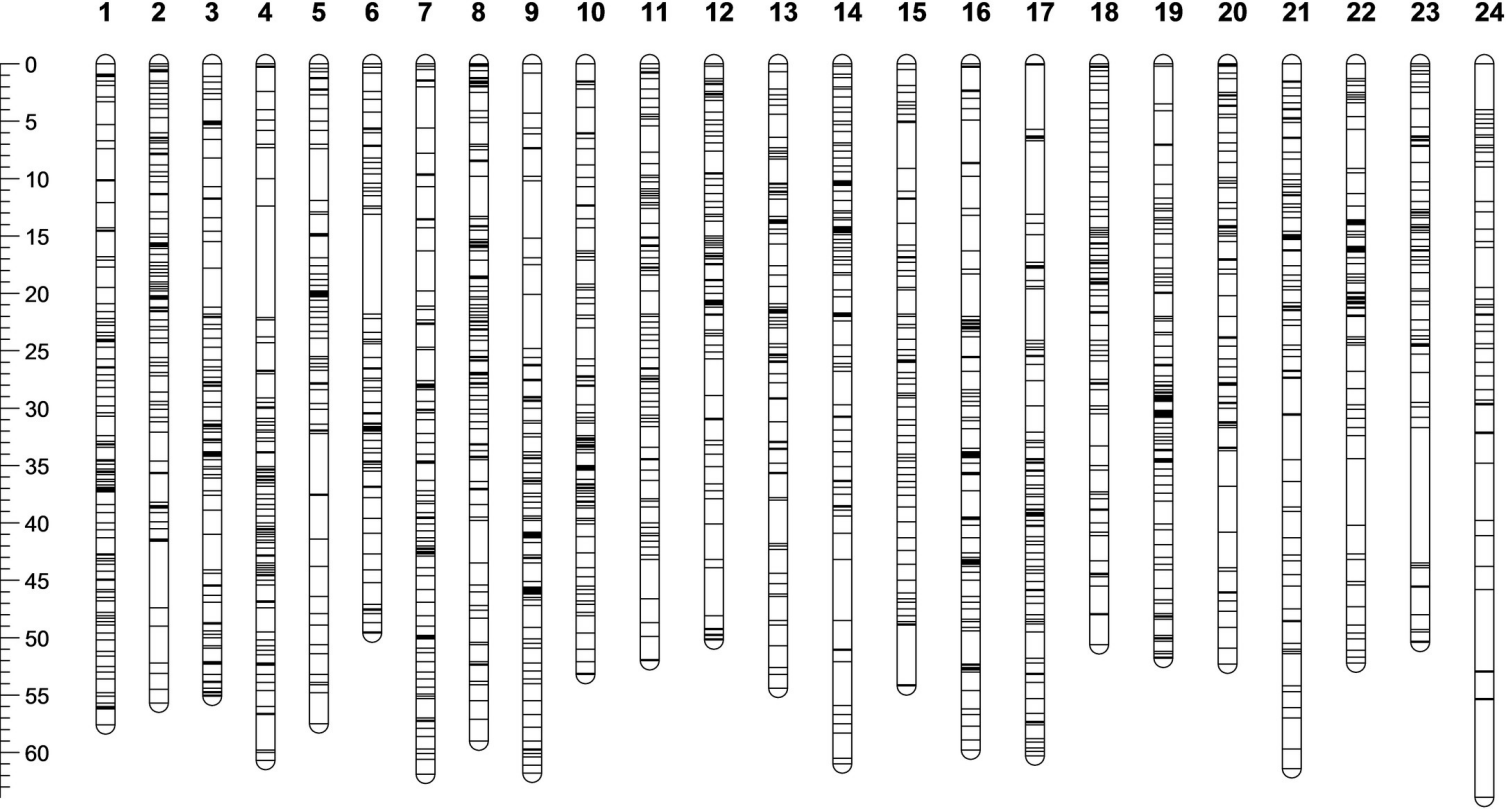
Diagram of the red snapper sex-averaged linkage map. This figure displays the marker distribution and linkage group sizes. A scale of map length in cM is presented on the left of the map for reference. Sex-averaged, male, and female map details are given in S3 Table.

Of the 10,150 markers (filtered SNPs and microsatellites) deemed suitable for mapping, 7,429, could initially be incorporated into 24 linkage groups (LGs), in agreement with the number of haploid chromosomes previously reported in several lutjanid species [39]. There were 6,556 maternally informative markers and 6,395 paternally informative markers and the number of genotypes available for linkage analysis per marker ranged between 92 and 290 (average 130.9). Removing error-prone markers identified in Lep-MAP reduced the number of loci included in the final map to 7,420. Sex-averaged linkage groups contained between 148 and 414 markers (average 309.17), and the average interval between adjacent markers was 0.18 cM (Table 2, Fig 1). The complete map spanned 1,346.64 cM, with LGs ranging in size from 49.64 to 63.89 cM (average 56.11 cM). The sex specific maps generated based on meioses observed in male and female parents spanned 1,322.42 cM and 1,449.83 cM respectively resulting in a ratio of 1.10:1 female to male map length (Table 2). Male LGs ranged in length from 43.15 to 72.7 cM (average 55.10 cM) while female LGs ranged from 48.49 to 75.59 cM (average 60.41).

**Table 2:** Summary statistics for the sex-averaged, male and female red snapper linkage map.

Sex-Averaged Map			Male Map	Female Map	
Linkage Group	Total Markers	cM	cM	cM	Female to Male Ratio
1	414	57.58	52.17	68.48	1.31
2	404	55.74	50.00	61.46	1.23
3	380	55.05	49.46	64.68	1.31
4	363	60.70	62.09	59.50	0.96
5	350	57.45	53.77	62.82	1.17
6	345	49.64	58.07	54.31	0.94
7	337	61.91	62.66	63.93	1.02
8	332	59.00	54.43	62.62	1.15
9	328	61.75	59.84	65.35	1.09
10	322	53.19	58.83	55.07	0.94
11	320	52.02	53.71	52.95	0.99
12	320	50.25	48.01	60.21	1.25
13	313	54.44	50.09	58.52	1.17
14	310	60.98	62.19	61.90	1.00
15	298	54.23	43.15	75.59	1.75
16	291	59.76	62.47	62.71	1.00
17	290	60.34	48.53	66.78	1.38
18	270	50.58	47.71	54.89	1.15
19	266	51.84	51.97	57.81	1.11
20	261	52.32	57.96	48.49	0.84
21	262	61.42	56.60	63.84	1.13
22	248	52.18	48.88	53.23	1.09
23	248	50.37	57.15	53.37	0.93
24	148	63.89	72.70	61.30	0.84
Total	7420	1,346.64	1,322.42	1,449.83	1.10

The magnitude of the difference in length between the male and female maps varied among linkage groups (average 1.10, range 0.84–1.75). Ratios close to one were most common, as shown in Table 2. One LG (15) displayed a ratio of 1.75, with female groups almost twice the length of corresponding male LG (Table 2).

A total of 5,883 contigs were positioned on the linkage map by ALLMAPS [3]. A majority of the mapped contigs (4,422) contained one SNP, and could in consequence not be oriented. The remaining 1,242 contigs included 2 or more markers and were tentatively oriented based on the mapping positions of their SNPs. The mapped contigs spanned more than 80.8 Mb, representing 10.5% of the draft genome assembly.

### Genome comparisons

**Fig 2:**
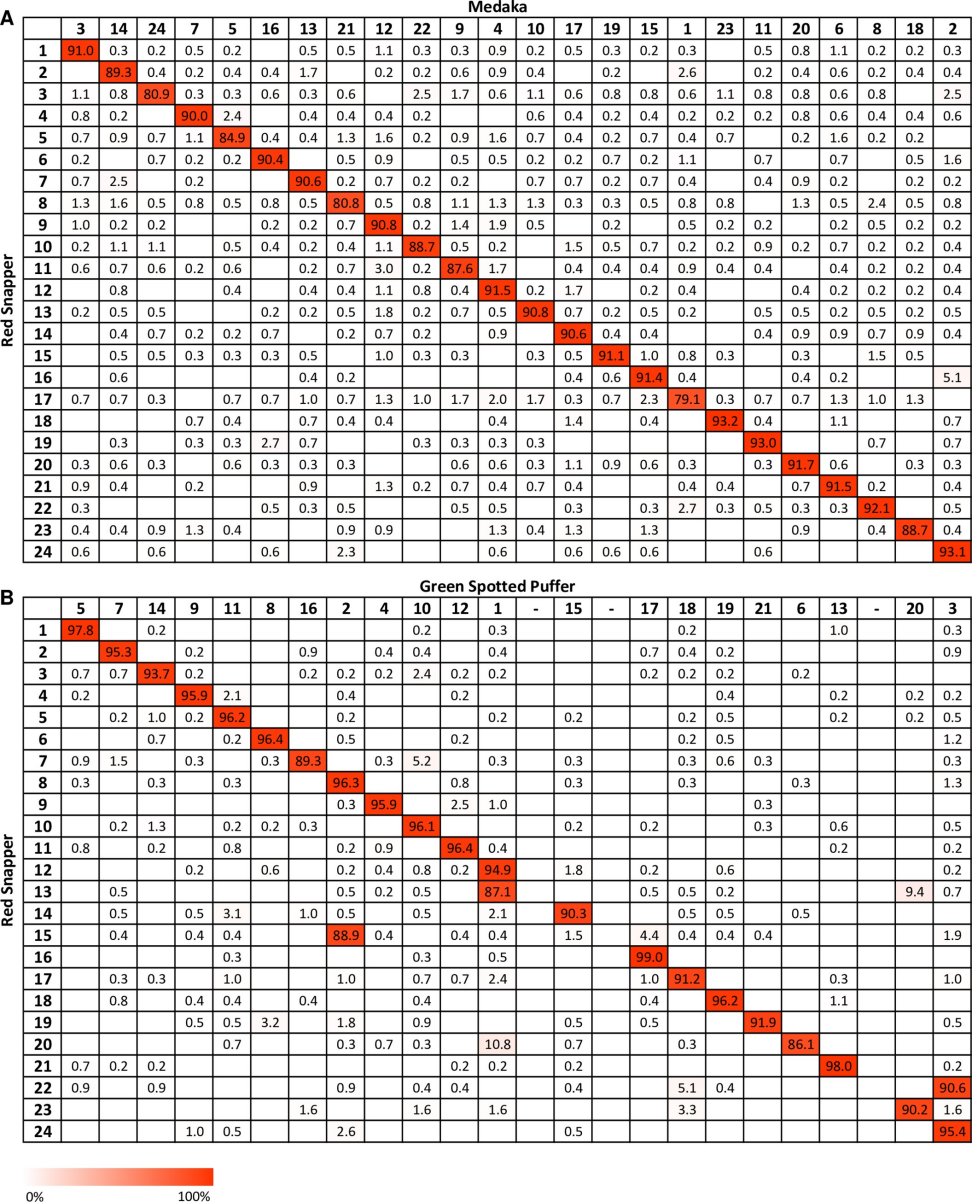
Oxford grids depicting syntenic regions between red snapper and two model species. Red snapper linkage groups are listed on the ordinate and the corresponding chromosomes of the comparison species are arranged on the abscissa. The percentage of red snapper sequences within a red snapper linkage group aligning to the chromosomes of the model species are given in the boxes of the Oxford grid. Comparisons between red snapper and (A) medaka (*Oryzias latipes*), (B) green spotted puffer (*Tetraodon nigroviridis*) are shown.

A total of 264 kb of repetitive elements were detected during screening of anchored genome contigs and these sequences were masked prior to performing genome comparisons. A total of 3.511 Mb (9,841 contig segments) and 2.883 Mb (8,720 contig segments) were aligned to unique sequences of the medaka, green spotted puffer and zebrafish reference genomes, respectively, using BLASTn. Each of the 24 red snapper linkage groups showed a high level of homology to one of the medaka chromosomes with, on average, 90.2% of sequences in one red snapper LG aligning to a unique medaka chromosome (range: 82.3% to 94.6%, Fig 2A). A high degree of synteny was also observed between red snapper and green spotted puffer (Fig 2B). Major differences between the two species can be attributed to the three fusion events that have occurred in the green spotted puffer lineage [40]. These chromosomal rearrangements gave rise to green spotted puffer chromosomes 1, 2 and 3 [40] and involved the ancestral chromosomes corresponding to current red snapper LGs 9 and 15, LGs 8 and 20 and LGs 23 and 24, respectively. On average, a higher percentage of sequences within a red snapper LG aligned to matching green spotted puffer chromosomes when compared to medaka (mean: 92.7%, range: 72.0% to 98.9%).

## Discussion

This work aimed to develop and characterize genomic resources for the red snapper *Lutjanus campechanus*. A high-density linkage map was generated and used to anchor contigs of a first draft genome sequence assembly, establishing, to our knowledge, the first draft reference genome for a lutjanid fish. The initial phase of the project focused on genome sequencing and assembly in order to establish reference genome contigs. The size of the red snapper genome was first estimated using the k-mer method which allowed assessing the fraction covered by the assembly. The estimate of genome size obtained in this study is slightly over 1.1 Gb which is smaller than the value (1.369 Gb) predicted from the published C-value for red snapper (1.4 pg, [41]). The latter C value estimate was obtained using a bulk fluorometric assay which is less accurate than current flow cytometry methods or Feulgen Densitometry. This estimate may also have been impacted by the use of different tissue types for the target species (red snapper, blood cells) and the standard (sea cucumber sperm). Estimates of C values for other lutjanids available in the animal genome size database [42] obtained using other methods are typically close to 1 or 1.1. These data thus suggest that the k-mer estimate obtained in this work may be a closer approximation of the actual size of the red snapper genome and this estimate is compared to the assembly span below. The assembly of short illumina reads and P-454 paired reads spans an estimated 69% of the red snapper genome and includes over 76 k contigs with a N50 value of 14 kb. Large numbers of contigs and a modest N50 value are common features of many first iterations of animal draft genome assemblies based primarily on short reads as this one. Factors impacting the efficiency of the assembly process include genome heterozygosity and the abundance of repetitive elements [2,43–45]. In teleosts, the proportion of repetitive DNA in the genome varies widely among species (range: 7–56%) and is positively correlated with genome size [46,47]. An empirical comparison of the genome sizes of several fish to their reported fractions of repetitive DNA indicates a very strong relationship between these two variables. These data suggest over 30% of the estimated 1,117 Mb red snapper genome may be composed of repetitive DNA. However, only 264 kb of repeats were detected during screening of mapped genome contigs, corresponding to approximately 0.33% of the portion of the assembly positioned on the map. The low representation of repetitive elements in the current assembly suggests that a potentially large fraction of the genomic data missing from the current assembly are repetitive elements and account for the high fragmentation observed in this work. Difficulties in assembling the red snapper genome are also likely due to the high heterozygosity typical of marine fishes and reported in all the previous genetic studies in this species [13,14].

High degrees of fragmentation are commonly reported in assemblies based solely on short sequencing reads (i.e. those produced via Illumina Sequencing by Synthesis technology). In this work, longer reads from paired-end P-454 pyrosequencing with two insert sizes (3 kb and 20 kb) were used in conjunction with short reads (2x100 bp Illumina sequencing of a 300 bp size selected library) in order to improve the assembly process [48], but the obtained hybrid dataset still yielded a large number of contigs of relatively small size. A main priority for the future development of the red snapper reference genome is to increase genome coverage and reduce fragmentation. The low coverage achieved during sequencing, in particular for the insert libraries, limited the assembly process and thus increasing coverage is a first priority. The production of long, continuous DNA sequencing reads (averaging 10–15 kb in length) using Pacific Biosciences (PacBio) Single Molecule Real-Time (SMRT) technology has proved critical for the correction of misassembled repetitive regions and for closing gaps in assemblies [49] and should also be incorporated in future sequencing efforts.

Considering the fragmented draft genome resulting from sequencing as discussed above, the chromosome location of genome contigs and scaffolds could only be determined through pairing the assembly to a high-density linkage map for use in interpretation of genome scans or in comparative studies of genome organization [5]. In this work, this pairing was achieved by using the genome contigs produced during the assembly as a reference to map RAD-sequencing reads generated during sequencing of parent and offspring members of mapping families. The SNPs discovered were in consequence directly mapped on the draft assembly allowing anchoring genome contigs onto linkage groups.

### Linkage map construction and characteristics

The linkage map of the red snapper produced during this study included 7,420 SNP and microsatellite loci distributed over 24 linkage groups. While there is no report on the number of chromosomes in red snapper to date, karyotypes produced in other lutjanids have consisted of 24 chromosome pairs [39], congruent with the finding of 24 linkage groups in red snapper. The sampling and filtering strategies used during construction of the map resulted in an average of 130 genotype available per locus per individual (range: 76–289), providing an average potential resolution of 0.76 cM (range: 0.34–1.09 cM). As this value exceeds the marker density at 0.18 cM, inferences on marker order are limited when distances between markers are smaller than the resolution achieved by the dataset.

Sex specific maps were generated based on partial datasets accounting for recombination observed in gametes of the male and female parents respectively. The female map was slightly longer than the male map with an average female to male map length ratio of 1.10:1. Discrepancies between male and female map lengths appear frequently in the literature, particularly among the fishes [50] and are attributed to sexually dimorphic patterns of recombination, a phenomenon termed heterochiasmy [51]. Relatively high female-to-male map length ratio have been reported in several other fish species, including Atlantic salmon (1.38:1) [52], silver carp (1.52:1) [53], European sea bass (1.60:1) [54,55] and rainbow trout (1.68:1) [55]. As discussed by Li et al. [56] theories proposed to explain recombination bias between sexes are multiple and include stronger selective pressures impacting male gametes [57], or selection acting on the efficacy of meiotic drive [58]. The female to male map length ratio in red snapper was in the low range of these values, only slightly above 1 more similar to findings in other marine species where this ratio was closer to 1 (see e.g. the turbot consensus map, 1.22 [59], the orange spotted grouper, 1.03 [60]) or even less than one (0.74 in the Mandarin fish [61]) and could reflect a reduced difference in selective pressure between gametes of the two sexes considering the high fecundity of females in marine fishes (typically in the order of several tens or hundreds thousands eggs per kg). The average female to male recombination ratio was not uniform across linkage groups, however. Female to male map length ratios ranged from 0.84:1 to 1.75:1 and 7 LGs (4, 6, 10, 11, 20, 23 and 24) showed ratios lower than 1.0. Female to male ratios varied widely among linkage groups in other species such as the channel catfish (0.97:1 to 2.50:1) [62] or the turbot 1.04:1 to 1.60:1 [59]. A practical consequence of differences between sexes in recombination rates for some genomic regions is that inferences based on sex-specific maps may be preferable when possible.

### Anchoring the draft genome

The linkage map was used to evaluate the initial assembly for potential errors. Misassembled contigs may occur in genomic regions rich in repetitive DNA, in particular when heterozygosity is high [44]. Detecting and resolving these assembly errors remains a central challenge in the production of draft genomes. Comparing assembled contigs to a high-density linkage map offers an independent measure of the assembly’s validity in that loci in proximal locations on genome contigs are expected to show near identical mapping position on the linkage map [2]. The consensus generated by allmaps did not show evidence of chimeric contigs mapping on different linkage groups or on distant positions on the linkage map.

### Genome comparisons

The integrated map and genome assembly was used to perform a comparative genomic analysis between red snapper and two model species, the medaka, and the green spotted puffer. While red snapper displayed synteny with both model species, the highest similarity was with medaka. The most recent common ancestor of medaka, green spotted puffer and zebrafish (hereafter referred to as the MTZ-ancestor) underwent 8 major chromosomal rearrangements, including 2 fissions, 4 fusions and 2 translocations within just 50 million years of the teleost-specific whole genome duplication event [40]. It is hypothesized that the original arrangement of the MTZ-ancestor has been preserved in medaka [40] which led to the proposal that medaka represents the evolutionarily basal chromosomal condition of the teleost genome, making it an informative comparison species. No major rearrangements were observed between medaka and red snapper, with each red snapper linkage group finding a strongly supported homologous chromosome in medaka (Fig 2). This high degree of synteny suggests that red snapper also shares the basal karyotype of the MTZ-ancestor.

Though red snapper displayed a higher degree of homology with medaka, linkage groups inferred in this study had stronger one-to-one relationships with green spotted puffer chromosomes: on average, higher proportions of red snapper sequences were found to be in common with green spotted puffer chromosomes, reflecting the closer phylogenetic relationship between green spotted puffer and red snapper relative to that of red snapper and medaka. Three fusion events have been proposed to have occurred since green spotted puffer split from medaka 184–198 million years ago, resulting in a haploid chromosome count of 21 [40]. Syntenic analyses between medaka and green spotted puffer show these fusions correspond to chromosomes 1, 2 and 3. Similar results were seen in red snapper (Fig 2) involving linkage groups orthologous to these three medaka chromosomes. Errors during linkage mapping and/or alignment of contigs to reference genomes, e.g. when sequence segments align to multiple regions of model species, likely account for the small fractions of red snapper chromosomes aligned to non-homologous chromosomes. Thus the synteny analysis will likely be enhanced when the red snapper draft genome is improved with the assembly of longer contigs.

## Conclusions

The integration of red snapper draft genome sequences and a high-density linkage map resulted in the production of first draft reference genome for a lutjanid, the red snapper *Lutjanus campechanus*. This resource will serve as a framework for future applied genomic studies in the red snapper and for expanding the resource towards a full reference for this species. The anchored draft sequence was applied in a comparative analysis of genome organization between red snapper and two model teleosts. The high degree of homology observed between red snapper and medaka supports the validity of the current draft assembly and suggests the hypothesized basal teleost karyotype is preserved in red snapper.

## Supporting information

S1 Table Characteristics of microsatellite multiplex assays: Composition of multiplex panels, fluorescent labels and thermal cycling protocols for multiplex assays. (XLSX)

S2 Table Results of genome sequencing and quality filtering. (XLSX)

S3 Table Details of the sex-averaged and sex-specific red snapper linkage maps, including marker ID, linkage group and genetic position in cM. (XLSX)

## References

[1] Ellegren H. Genome sequencing and population genomics in non-model organisms. Trends Ecol Evol (Amst). 2014;29: 51–63. 10.1016/j.tree.2013.09.008 24139972

[2] Fierst JL. Using linkage maps to correct and scaffold de novo genome assemblies: methods, challenges, and computational tools. Front Genet. 2015;6: 220 10.3389/fgene.2015.00220 26150829 PMC4473057

[3] Tang H, Zhang X, Miao C, Zhang J, Ming R, Schnable JC, et al ALLMAPS: robust scaffold ordering based on multiple maps. Genome Biol. 2015;16: 3 10.1186/s13059-014-0573-1 25583564 PMC4305236

[4] Hedgecock D, Shin G, Gracey AY, Den Berg DV, Samanta MP. Second-Generation Linkage Maps for the Pacific Oyster Crassostrea gigas Reveal Errors in Assembly of Genome Scaffolds. G3 (Bethesda). 2015;5: 2007–2019. 10.1534/g3.115.019570 26248981 PMC4592983

[5] Shao C, Niu Y, Rastas P, Liu Y, Xie Z, Li H, et al Genome-wide SNP identification for the construction of a high-resolution genetic map of Japanese flounder (Paralichthys olivaceus): applications to QTL mapping of Vibrio anguillarum disease resistance and comparative genomic analysis. DNA Res. 2015;22: 161–170. 10.1093/dnares/dsv001 25762582 PMC4401326

[6] Kai W, Nomura K, Fujiwara A, Nakamura Y, Yasuike M, Ojima N, et al A ddRAD-based genetic map and its integration with the genome assembly of Japanese eel (Anguilla japonica) provides insights into genome evolution after the teleost-specific genome duplication. BMC Genomics. 2014;15: 233 10.1186/1471-2164-15-233 24669946 PMC3986909

[7] Hillier LW, Miller RD, Baird SE, Chinwalla A, Fulton LA, Koboldt DC, et al Comparison of C. elegans and C. briggsae genome sequences reveals extensive conservation of chromosome organization and synteny. PLoS Biol. 2007;5: e167 10.1371/journal.pbio.0050167 17608563 PMC1914384

[8] Bourret V, Kent MP, Primmer CR, Vasemägi A, Karlsson S, Hindar K, et al SNP-array reveals genome-wide patterns of geographical and potential adaptive divergence across the natural range of Atlantic salmon (Salmo salar). Mol Ecol. 2013;22: 532–551. 10.1111/mec.12003 22967111

[9] Allendorf FW, Luikart G. Conservation And The Genetics Of Populations. 1st ed. Wiley-blackwell; 2006 p. 664.

[10] Hollenbeck CM, Portnoy DS, Gold JR. A method for detecting recent changes in contemporary effective population size from linkage disequilibrium at linked and unlinked loci. Heredity. 2016;117: 207–216. 10.1038/hdy.2016.30 27165767 PMC5026753

[11] Yue GH. Recent advances of genome mapping and marker-assisted selection in aquaculture. Fish and Fisheries. 2014;15: 376–396. 10.1111/faf.12020

[12] Pruett CL, Saillant E, Gold JR. Historical population demography of red snapper (Lutjanus campechanus) from the northern Gulf of Mexico based on analysis of sequences of mitochondrial DNA. Mar Biol. 2005;147: 593–602. 10.1007/s00227-005-1615-8

[13] Saillant E, Bradfield SC, Gold JR. Genetic variation and spatial autocorrelation among young-of-the-year red snapper (Lutjanus campechanus) in the northern Gulf of Mexico. ICES Journal of Marine Science. 2010;67: 1240–1250. 10.1093/icesjms/fsq011

[14] Gold JR, Saillant E. Population structure of red snapper in the northern Gulf of Mexico. American Fisheries Society Symposium. American Fisheries Society, 5410 Grosvenor Ln. Ste. 110 Bethesda MD 20814–2199 USA,; 2007. pp. 181–195. Available: https://www.researchgate.net/profile/John_Gold3/publication/255571204_Population_Structure_of_Red_Snapper_in_the_Northern_Gulf_of_Mexico/links/551569280cf2d70ee2702484.pdf

[15] Woods MK, Fischer AJ, Jr JC, Nieland DL. Size and age at maturity of female red snapper Lutjanus campechanus in the northern Gulf of Mexico. 2003;

[16] Fischer AJ, Jr MB, Wilson CA. Red snapper (Lutjanus campechanus) demographic structure in the northern Gulf of Mexico based on spatial patterns in growth rates and morphometrics. Fishery Bulletin. 2004;

[17] Puritz JB, Gold JR, Portnoy DS. Fine-scale partitioning of genomic variation among recruits in an exploited fishery: causes and consequences. Sci Rep. 2016;6: 36095 10.1038/srep36095 27782185 PMC5080595

[18] Marçais G, Kingsford C. A fast, lock-free approach for efficient parallel counting of occurrences of k-mers. Bioinformatics. 2011;27: 764–770. 10.1093/bioinformatics/btr011 21217122 PMC3051319

[19] Myers EW, Sutton GG, Delcher AL, Dew IM, Fasulo DP, Flanigan MJ, et al A whole-genome assembly of Drosophila. Science. 2000;287: 2196–2204. 10.1126/science.287.5461.2196 10731133

[20] Miller JR, Delcher AL, Koren S, Venter E, Walenz BP, Brownley A, et al Aggressive assembly of pyrosequencing reads with mates. Bioinformatics. 2008;24: 2818–2824. 10.1093/bioinformatics/btn548 18952627 PMC2639302

[21] Gilchrist AS, Shearman DCA, Frommer M, Raphael KA, Deshpande NP, Wilkins MR, et al The draft genome of the pest tephritid fruit fly Bactrocera tryoni: resources for the genomic analysis of hybridising species. BMC Genomics. 2014;15: 1153 10.1186/1471-2164-15-1153 25527032 PMC4367827

[22] Minton V, Hawke J, Tatum W. Hormone induced spawning of red snapper, *Lutjanus campechanus*. Aquaculture. 1983;30: 363–368.

[23] Sambrook J, Fritsch E f., Maniatis T. Molecular Cloning: A Laboratory Manual (3 Volume Set). 2nd ed. Cold Spring Harbor Laboratory Pr; 1987 p. 3.

[24] Norrell AE, Crawley D, Jones KL, Saillant EA. Development and characterization of eighty-four microsatellite markers for the red snapper (Lutjanus campechanus) using Illumina paired-end sequencing. Aquaculture. 2014;430: 128–132. 10.1016/j.aquaculture.2014.04.005

[25] Gold JR, Pak E, Richardson LR. Microsatellite variation among red snapper (Lutjanus campechanus) from the Gulf of Mexico. Mar Biotechnol. 2001;3: 293–304. 10.1007/s10126-001-0004-7 14961368

[26] Renshaw MA, Saillant E, Bradfield SC, Gold JR. Microsatellite multiplex panels for genetic studies of three species of marine fishes: red drum (Sciaenops ocellatus), red snapper (Lutjanus campechanus), and cobia (Rachycentron canadum). Aquaculture. 2006;253: 731–735. 10.1016/j.aquaculture.2005.09.012

[27] Danzmann RG. LINKMFEX: linkage analysis software for diploid and tetraploid outcrossed mapping panels. Aquaculture. 2005;

[28] Baird NA, Etter PD, Atwood TS, Currey MC, Shiver AL, Lewis ZA, et al Rapid SNP discovery and genetic mapping using sequenced RAD markers. PLoS One. 2008;3: e3376 10.1371/journal.pone.0003376 18852878 PMC2557064

[29] Peterson BK, Weber JN, Kay EH, Fisher HS, Hoekstra HE. Double digest RADseq: an inexpensive method for de novo SNP discovery and genotyping in model and non-model species. PLoS One. 2012;7: e37135 10.1371/journal.pone.0037135 22675423 PMC3365034

[30] Schweyen H, Rozenberg A, Leese F. Detection and removal of PCR duplicates in population genomic ddRAD studies by addition of a degenerate base region (DBR) in sequencing adapters. Biol Bull. 2014;227: 146–160. 10.1086/BBLv227n2p146 25411373

[31] Puritz JB, Hollenbeck CM, Gold JR. dDocent: a RADseq, variant-calling pipeline designed for population genomics of non-model organisms. PeerJ. 2014;2: e431 10.7717/peerj.431 24949246 PMC4060032

[32] Li H. Aligning sequence reads, clone sequences and assembly contigs with BWA-MEM. arXiv:13033997 [q-bio]. 2013; Available: http://arxiv.org/abs/1303.3997

[33] Garrison E, Marth G. Haplotype-based variant detection from short-read sequencing. arXiv:12073907 [q-bio]. 2012; Available: http://arxiv.org/abs/1207.3907

[34] Rastas P, Paulin L, Hanski I, Lehtonen R, Auvinen P. Lep-MAP: fast and accurate linkage map construction for large SNP datasets. Bioinformatics. 2013;29: 3128–3134. 10.1093/bioinformatics/btt563 24078685 PMC4433499

[35] Rastas P, Calboli FCF, Guo B, Shikano T, Merilä J. Construction of Ultradense Linkage Maps with Lep-MAP2: Stickleback F2 Recombinant Crosses as an Example. Genome Biol Evol. 2015;8: 78–93. 10.1093/gbe/evv250 26668116 PMC4758246

[36] Kosambi DD. The estimation of map distances from recombination values. Ann Eugen. 1943;12: 172–175. 10.1111/j.1469-1809.1943.tb02321.x

[37] Voorrips RE. MapChart: software for the graphical presentation of linkage maps and QTLs. J Hered. 2002;93: 77–78. 10.1093/jhered/93.1.77 12011185

[38] Smit AFA, Hubley R, Green P. RepeatMasker Open-4.0. 2013–2015. 2015;

[39] Rocha ÉC, Molina WF. Cytogenetic analysis in western Atlantic snappers (Perciformes, Lutjanidae). Genetics and Molecular Biology. 2008;31: 461–467.

[40] Kasahara M, Naruse K, Sasaki S, Nakatani Y, Qu W, Ahsan B, et al The medaka draft genome and insights into vertebrate genome evolution. Nature. 2007;447: 714–719. 10.1038/nature05846 17554307

[41] Hinegardner R, Rosen DE. Cellular DNA content and the evolution of teleostean fishes. Am Nat. 1972;106: 621–644. 10.1086/282801

[42] Gregory TR. Animal genome size database. http://www.genomesize.com. 2002;

[43] Miller JR, Koren S, Sutton G. Assembly algorithms for next-generation sequencing data. Genomics. 2010;95: 315–327. 10.1016/j.ygeno.2010.03.001 20211242 PMC2874646

[44] Treangen TJ, Salzberg SL. Repetitive DNA and next-generation sequencing: computational challenges and solutions. Nat Rev Genet. 2011;13: 36–46. 10.1038/nrg3117 22124482 PMC3324860

[45] Vinson JP, Jaffe DB, O’Neill K, Karlsson EK, Stange-Thomann N, Anderson S, et al Assembly of polymorphic genomes: algorithms and application to Ciona savignyi. Genome Res. 2005;15: 1127–1135. 10.1101/gr.3722605 16077012 PMC1182225

[46] Yuan Z, Liu S, Zhou T, Tian C, Bao L, Dunham R, et al Comparative genome analysis of 52 fish species suggests differential associations of repetitive elements with their living aquatic environments. BMC Genomics. 2018;19: 141 10.1186/s12864-018-4516-1 29439662 PMC5811955

[47] Volff JN. Genome evolution and biodiversity in teleost fish. Heredity. 2005;94: 280–294. 10.1038/sj.hdy.6800635 15674378

[48] Schatz MC, Delcher AL, Salzberg SL. Assembly of large genomes using second-generation sequencing. Genome Res. 2010;20: 1165–1173. 10.1101/gr.101360.109 20508146 PMC2928494

[49] Richards S, Murali SC. Best practices in insect genome sequencing: what works and what doesn’t. Curr Opin Insect Sci. 2015;7: 1–7. 10.1016/j.cois.2015.02.013 26085980 PMC4465116

[50] Liu S, Rexroad CE, Couch CR, Cordes JF, Reece KS, Sullivan CV. A microsatellite linkage map of striped bass (Morone saxatilis) reveals conserved synteny with the three-spined stickleback (Gasterosteus aculeatus). Mar Biotechnol. 2012;14: 237–244. 10.1007/s10126-011-9407-2 21968826

[51] Burt A, Bell G, Harvey PH. Sex differences in recombination. J Evol Biol. 1991;4: 259–277.

[52] Lien S, Gidskehaug L, Moen T, Hayes BJ, Berg PR, Davidson WS, et al A dense SNP-based linkage map for Atlantic salmon (Salmo salar) reveals extended chromosome homeologies and striking differences in sex-specific recombination patterns. BMC Genomics. 2011;12: 615 10.1186/1471-2164-12-615 22182215 PMC3261913

[53] Guo W, Tong J, Yu X, Zhu C, Feng X, Fu B, et al A second generation genetic linkage map for silver carp (Hypophthalmichehys molitrix) using microsatellite markers. Aquaculture. 2013;412–413: 97–106. 10.1016/j.aquaculture.2013.06.027

[54] Chistiakov DA, Hellemans B, Haley CS, Law AS, Tsigenopoulos CS, Kotoulas G, et al A microsatellite linkage map of the European sea bass Dicentrarchus labrax L. Genetics. 2005;170: 1821–1826. 10.1534/genetics.104.039719 15937133 PMC1449790

[55] Rexroad CE, Palti Y, Gahr SA, Vallejo RL. A second generation genetic map for rainbow trout (Oncorhynchus mykiss). BMC Genet. 2008;9: 74 10.1186/1471-2156-9-74 19019240 PMC2605456

[56] Li X, Li L, Yan J. Dissecting meiotic recombination based on tetrad analysis by single-microspore sequencing in maize. Nat Commun. 2015;6: 6648 10.1038/ncomms7648 25800954 PMC4383000

[57] Lenormand T, Dutheil J. Recombination difference between sexes: a role for haploid selection. PLoS Biol. 2005;3: e63 10.1371/journal.pbio.0030063 15736976 PMC1044830

[58] Brandvain Y, Coop G. Scrambling eggs: meiotic drive and the evolution of female recombination rates. Genetics. 2012;190: 709–723. 10.1534/genetics.111.136721 22143919 PMC3276612

[59] Maroso F, Hermida M, Millán A, Blanco A, Saura M, Fernández A, et al Highly dense linkage maps from 31 full-sibling families of turbot (Scophthalmus maximus) provide insights into recombination patterns and chromosome rearrangements throughout a newly refined genome assembly. DNA Res. 2018;25: 439–450. 10.1093/dnares/dsy015 29897548 PMC6105115

[60] You X, Shu L, Li S, Chen J, Luo J, Lu J, et al Construction of high-density genetic linkage maps for orange-spotted grouper Epinephelus coioides using multiplexed shotgun genotyping. BMC Genet. 2013;14: 113 10.1186/1471-2156-14-113 24289265 PMC3890575

[61] Sun C, Niu Y, Ye X, Dong J, Hu W, Zeng Q, et al Construction of a high-density linkage map and mapping of sex determination and growth-related loci in the mandarin fish (Siniperca chuatsi). BMC Genomics. 2017;18: 446 10.1186/s12864-017-3830-3 28587594 PMC5461734

[62] Li Y, Liu S, Qin Z, Waldbieser G, Wang R, Sun L, et al Construction of a high-density, high-resolution genetic map and its integration with BAC-based physical map in channel catfish. DNA Res. 2015;22: 39–52. 10.1093/dnares/dsu038 25428894 PMC4379976

